# Comparison of School-Based and Community-Wide Mass Drug Administration for Schistosomiasis Control in an Area of Western Kenya with High Initial *Schistosoma mansoni* Infection Prevalence: A Cluster Randomized Trial

**DOI:** 10.4269/ajtmh.19-0626

**Published:** 2019-12-02

**Authors:** W. Evan Secor, Ryan E. Wiegand, Susan P. Montgomery, Diana M. S. Karanja, Maurice R. Odiere

**Affiliations:** 1 Division of Parasitic Diseases and Malaria, Centers for Disease Control and Prevention, Atlanta, Georgia;; 2 Center for Global Health Research, Kenya Medical Research Institute, Kisumu, Kenya

## Abstract

We conducted a cluster randomized trial comparing the target population and timing of mass drug administration (MDA) with praziquantel for control of schistosomiasis in villages in western Kenya with high initial prevalence (> 25%) according to a harmonized protocol developed by the Schistosomiasis Consortium for Operational Research and Evaluation. A total of 150 villages were randomized into six treatment arms (25 villages per arm), were assessed at baseline, and received two or four rounds of MDA using community-wide (CWT) or school-based (SBT) treatment over 4 years. In the fifth year, a final evaluation was conducted. The primary outcomes were prevalence and intensity of *Schistosoma mansoni* infections in children aged 9–12 years, each year their village received MDA. Baseline and year 5 assessments of first-year students and adults were also performed. Using Poisson and negative binomial regression with generalized estimating equations, we found similar effects of CWT and SBT MDA treatment strategies in children aged 9–12 years: significant reductions of prevalence of infection in all arms and of heavy-intensity (≥ 400 eggs/gram) infections in most arms but no significant differences between arms. Combined arms of villages that received four rounds of treatment had greater reduction than villages in arms that only received two rounds of treatment. Surprisingly, we also found benefits of SBT for first-year primary students and adults, who never received treatment in those arms. Our data support the use of annual SBT for control programs when coupled with attention to infections in younger children and occasional treatment of adults.

## INTRODUCTION

Mass drug administration (MDA) is a proven strategy for lymphatic filariasis, onchocerciasis, blinding trachoma, soil-transmitted helminths, and schistosomiasis control programs. By providing regular MDA to at-risk populations, infection levels of these neglected tropical diseases (NTDs) can be reduced to a point where the force of transmission is reduced and morbidity is moderated.^1^ Because the treatments used are safe for uninfected individuals, in most settings, the program expenditures for providing MDA to the whole at-risk population are more cost effective than diagnosing and treating each infected individual.

The programs for different NTDs are at various stages of maturation. Whereas lymphatic filariasis and blinding trachoma have fairly well-defined strategies for where to initiate MDA, how to track progress, and when treatment can be stopped,^2,3^ other programs such as schistosomiasis control still have important operational research questions that require attention. This is in part due to the longer time that donated drugs used for treating lymphatic filariasis (ivermectin and albendazole) and trachoma (azithromycin) have been available. However, in recent years, generous donations from pharmaceutical companies, governmental bodies, and private foundations have made it possible to begin widespread MDA for schistosomiasis using praziquantel.

In part to address the unanswered questions about best methods for schistosomiasis control, the Schistosomiasis Consortium for Operational Research and Evaluation (SCORE) was formed.^4^ A primary goal of SCORE has been to identify the most effective strategies for reducing prevalence and intensity of schistosome infections in endemic areas with different initial prevalence of *Schistosoma mansoni* or *Schistosoma haematobium* through randomized trials to compare MDA frequency and target population. We conducted one of the SCORE studies in 150 villages in western Kenya with high baseline prevalence (> 25%) of *S. mansoni*. Over a 5-year period, we compared the impact of MDA provided at the community or school level either annually or with intervening years. Assessments of infection prevalence and intensity in children aged 9–12 years were performed at baseline, each year MDA was provided, and a final evaluation was conducted. The studies focused on school-aged children (SAC) because this is the age-group that typically has the highest infection levels. In addition, infection levels in community adults and first-year students were assessed at baseline and the final evaluation to determine what effect MDA may have on infection levels in the larger community.

## METHODS

### Ethical approval.

This study was reviewed and approved by the Scientific and Ethics Review Committees (ERCs) of the Kenya Medical Research Institute (KEMRI, SSC #1820); the Institutional Review Board (IRB) of the CDC (protocol #6016) relied on the KEMRI ERC. Administrative review and approval was also provided by the IRB of the University of Georgia. Written informed consent was obtained each year of the study from participants or guardians of participants younger than 18 years, and assent was obtained from children. The trial was registered with the ISRCTN (#16755535).

### Study design.

**Figure 1.**
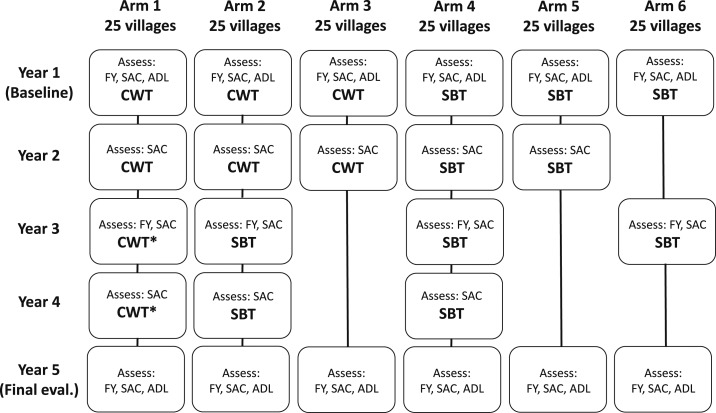
Diagram of trial design by arm and by year. First-year students (FYs), school-aged children (SAC), or adults (ADL) were assessed each year, and mass drug administration was provided through community-wide treatment (CWT) or school-based treatment (SBT) in years 1 through 4. A final evaluation was conducted in year 5.

The study was a cluster randomized trial carried out according to the previously published harmonized protocol for gaining control developed by the SCORE investigators.^4^ In brief, villages were eligible for inclusion if at least 25% of 50 sampled 13- and 14-year-olds were positive for *S. mansoni* eggs by the Kato–Katz method in at least one of two fecal smear slides prepared from a single stool sample. The 150 eligible villages were then randomized into one of 6 treatment arms of 25 villages each (Figure 1). In arm 1, community-wide treatment (CWT) was provided annually for 4 years; arm 2 villages received CWT for 2 years and school-based treatment (SBT) for 2 years; and CWT was conducted in arm 3 villages for the first two years only. In arm 4, SBT was provided each year for 4 years; arm 5 villages received SBT for the first 2 years only; and in arm 6, SBT was distributed every other year. Praziquantel treatment was provided using dose poles to approximate 40 mg drug per kg body weight. Community-wide treatment was performed by community drug distributors, who went house to house to treat all eligible members of the community. School-based treatment was distributed by health teachers to all primary school students. Before this study, no MDAs with praziquantel had been conducted in this area.

In each village, infection prevalence and intensity were measured in a targeted sample of 100 children between the ages of nine and 12 years at baseline (year 1), each treatment year, and at final evaluation (year 5). Sample collection from children took place at a village’s primary school. If there were more than 100 children eligible for inclusion in a village, enrollment records were used to generate a random list to recruit participants. Children not attending school were also invited to come to schools on sampling and MDA days. However, because primary education is free in Kenya and > 95% of the eligible population attends school, the sample was effectively from all school-aged children.

Participating children were assigned a unique identification number and provided with a corresponding barcoded stool collection cup each morning over 3 consecutive days. Returned stool samples were used to prepare two slides per stool according to the Kato–Katz method. Thus, up to six slides were prepared per student, although not every student was able to produce a stool sample on every day. Trained microscopists enumerated the number of eggs per slide and calculated the eggs per gram feces (EPG). For the estimation of prevalence, a child was considered positive if a schistosome egg was identified on any slide. Infection intensity was the average EPG from all slides examined for a given individual and was categorized as light (< 100 EPG), moderate (100–399 EPG), or heavy (≥ 400 EPG) according to WHO guidelines.^5^

In each village, at years 1 and 5, three stool samples were also sought from 100 first-year students as was a single stool sample from 50 adults aged between 20 and 55 years. First-year students were recruited in the same way as 9–12-year-old children and sampled at primary schools. Adults were recruited at their homes with the assistance of community health workers and village elders using a random sample of census data.

### Coverage.

All treatments were recorded in treatment booklets to determine the reported treatment coverage. Coverage for SBT was estimated by dividing the number of children who received treatment at the school by the total school enrollment, with a target of treating at least 90% of the students. For CWT, the number of people who received treatment was compared with the village census, with a targeted coverage of ≥ 75%.

### Sample size calculation.

Sample size calculations are detailed by Ezeamama et al.^4^ In brief, sample size calculation assumed a binomial model with generalized estimating equations (GEEs). A baseline prevalence of 50% was assumed for all arms. For a two-sided test with a 5% level of significance, 25 villages per arm and 100 children per village provide 90% power to detect a difference between a target level of 15% for the most intense treatment arm and an absolute difference of 11.4%. These calculations assumed an over-dispersion parameter of 5.0 and a trivial correlation between observations at years 1 and 5.

### Statistical analyses.

All analyses were performed at the individual level, used the 5% level of significance, used SAS version 9.3 (SAS Institute, Inc., Cary, NC), and accounted for community-level clustering of infections. Basic summary statistics (e.g., mean prevalence and intensity) accounted for village-level clustering via Taylor series linearization.^6^ Comparisons between arms were performed with negative binomial regression with GEEs, with an exchangeable correlation structure.^7^ Results are reported as a prevalence ratio (PR) and 95% CI when analyzing *S. mansoni* infection prevalence or an arithmetic mean ratio (AMR) and 95% CI when analyzing *S. mansoni* infection intensity in EPG. Adjusted models controlled for age and gender. Comparisons between individual arms assessed the change from year 1 to year 5 to account for differences at baseline. In the analyses comparing four and two treatments, summary statistics indicated similar prevalence at year 1, thus only year 5 was evaluated. Mixed models were used to calculate interclass correlation coefficients and design effects.^8,9^ All results from regression models reported in the text control for age and gender.

Aforementioned models were also fit without villages contained within a hotspot of elevated infection found in spatial analyses of the data.^10^ These models were implemented with the same methodology described earlier.

## RESULTS

### Effect of different MDA approaches.

**Figure 2.**
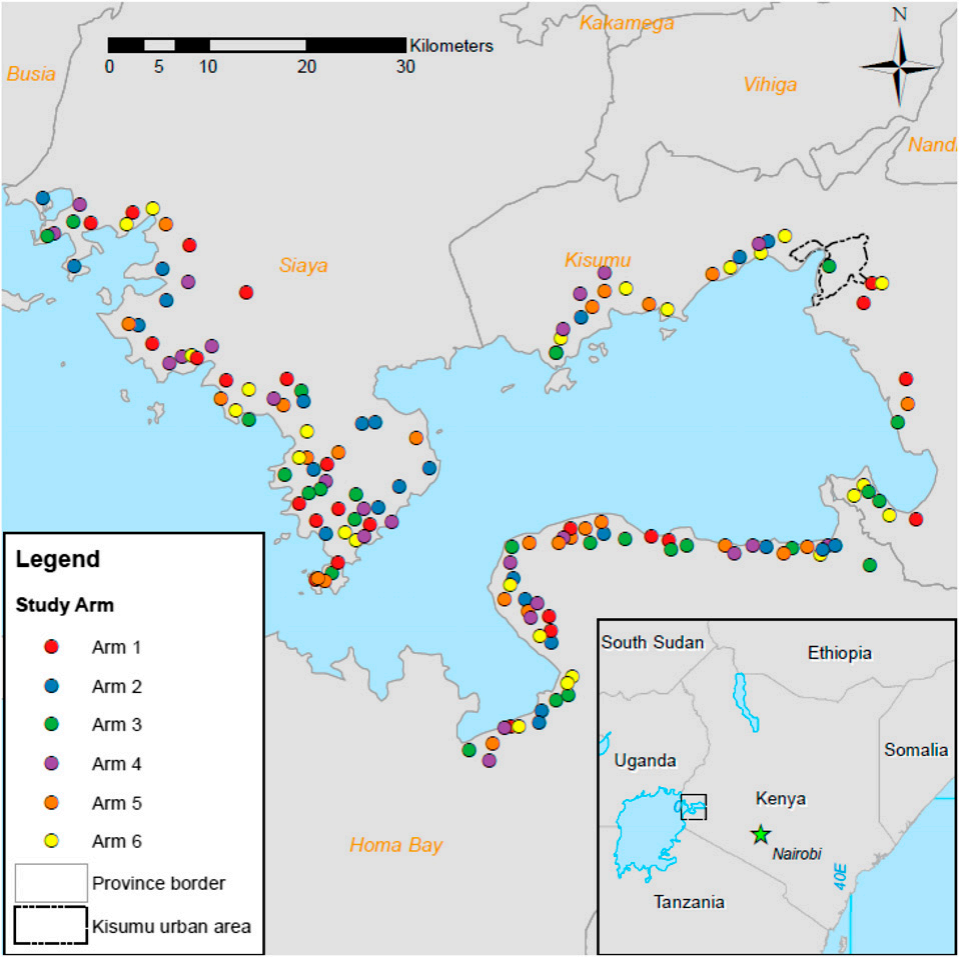
Map showing locations of villages in each arm. Inset shows location of study area in western Kenya. This figure appears in color at www.ajtmh.org.

**Figure 3.**
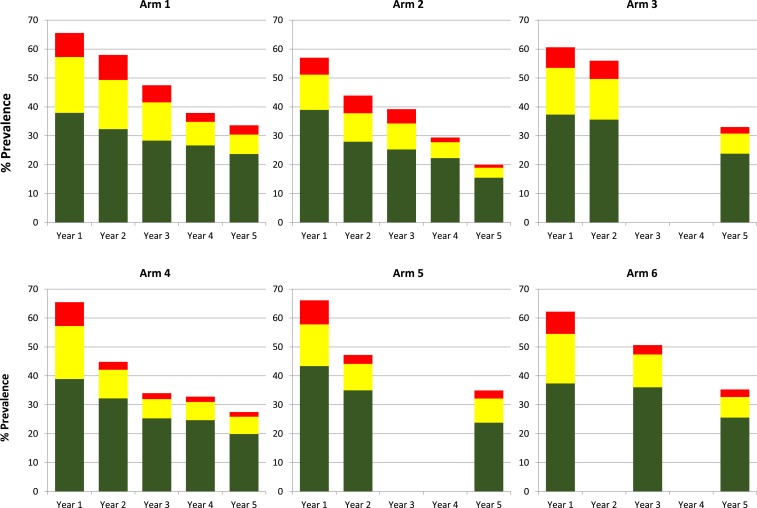
Overall mean prevalence and mean prevalence of light (green), moderate (yellow), and heavy (red) intensity infections by arm and year for 9- to 12-year-old children. Each arm consists of 25 villages. This figure appears in color at www.ajtmh.org.

Assessments for inclusion were conducted in 358 villages to identify 150 eligible villages that were randomized into six arms of 25 villages each (Figure 2). Numbers of children aged 9–12 years who provided samples in each arm at years 1 and 5 are shown in Table 1 (participant numbers for years 2–4 are available in Supplemental Table 1). Overall prevalence and prevalence of heavy- (≥ 400 EPG), moderate- (100–399 EPG), and light-intensity (1–99 EPG) infections were calculated for each year stool data collected (Figure 3). In all six arms, infection prevalence was significantly less at year 5 than year 1 (*P* ≤ 0.001, Table 2). Prevalence of heavy-intensity infections also decreased over time, with four arms demonstrating significantly reduced levels by year 5. Also, all arms demonstrated < 5% mean prevalence of heavy-intensity infections (Table 1), the current WHO definition of morbidity control. However, there were no significant differences between arms, with SBT arms showing similar proportional decreases in infection prevalence and intensity as CWT arms (Supplemental Table 2). Treatment coverage data are shown in Table 3. In year 1, average arm coverage for SAC was lower than the 90% target for all arms, with average coverage in CWT arms (range 69.3–74.7%) lower than that of SBT arms (83.0–86.4%). In year 2, average coverage for SBT arms achieved the desired SAC target treatment, whereas average coverage for 2 of the 3 CWT arms remained < 80%. In the harmonized SCORE protocol, arms 1–3 were meant to include provision of SBT along with CWT. However, we misinterpreted this in years 1 and 2 and provided only CWT consisting of house-to-house treatment, including school-aged children. In years 3 and 4, CWT included both the house-to-house treatment and SBT in primary schools. In years 3 and 4, communities scheduled for CWT that also received SBT still failed to reach 90% average SAC coverage, whereas communities scheduled for SBT consistently had ≥ 89% average SAC coverage. Average coverage of the total population in communities that received CWT was consistently above the 75% target.

**Table 1:** Prevalence and intensity summary statistics for 9- to 12-year-olds by arm at baseline and final evaluation

		Year 1		Year 5	
Variable	Arm	*n*/*N* or *N*	% (CI), mean (CI), or median (range)	*n*/*N* or *N*	% (CI), mean (CI), or median (range)
Village prevalence (mean % infected, CI)	Arm 1	1,176/1794	65.55 (56.02–75.09)	775/2,294	33.78 (21.72–45.83)
	Arm 2	1,113/1951	57.05 (49.35–64.75)	456/2,274	20.05 (10.23–29.88)
	Arm 3	1,233/2035	60.59 (51.35–69.83)	775/2,344	33.06 (23.57–42.55)
	Arm 4	1,193/1822	65.48 (55.73–75.23)	595/2,165	27.48 (17.05–37.92)
	Arm 5	1,274/1927	66.11 (57.66–74.56)	820/2,345	34.97 (25.06–44.87)
	Arm 6	1,251/2012	62.18 (53.20–71.15)	841/2,385	35.26 (24.94–45.58)
Prevalence of heavy intensity infections (village mean % eggs per gram feces ≥ 400, CI)	Arm 1	149/1794	8.31 (4.15–12.46)	73/2,294	3.18 (0.00–6.47)
	Arm 2	114/1951	5.84 (1.47–10.22)	24/2,274	1.06 (0.00–2.49)
	Arm 3	144/2035	7.08 (3.51–10.64)	53/2,344	2.26 (1.08–3.44)
	Arm 4	150/1822	8.23 (3.62–12.85)	35/2,165	1.62 (0.55–2.69)
	Arm 5	160/1927	8.30 (2.69–13.92)	65/2,345	2.77 (0.11–5.43)
	Arm 6	154/2012	7.65 (2.90–12.41)	62/2,385	2.60 (0.00–5.53)
Village intensity (mean all participants, CI)	Arm 1	1,794	104.67 (65.00–144.34)	2,294	47.77 (11.34–84.20)
	Arm 2	1,951	74.34 (36.91–111.78)	2,274	19.15 (0.00–38.85)
	Arm 3	2,035	91.65 (59.80–123.49)	2,344	39.14 (20.63–57.64)
	Arm 4	1,822	100.76 (59.39–142.12)	2,165	30.16 (12.98–47.33)
	Arm 5	1,927	97.89 (47.94–147.84)	2,345	45.95 (11.81–80.09)
	Arm 6	2,012	153.95 (102.27–205.64)	2,385	45.10 (8.60–81.60)

Each arm consists of 25 villages.

**Table 2:** Comparisons of change in prevalence in 9- to 12-year-olds and intensity of *Schistosoma mansoni* infection between year 1 and year 5 for each arm

	Prevalence			
Arm	Crude PR (CI)	*P*-value	Adjusted PR (CI)	*P*-value
1	0.51 (0.34, 0.76)	< 0.001	0.49 (0.34, 0.73)	< 0.001
2	0.34 (0.21, 0.56)	< 0.001	0.34 (0.21, 0.54)	< 0.001
3	0.53 (0.38, 0.73)	< 0.001	0.59 (0.44, 0.80)	< 0.001
4	0.42 (0.28, 0.64)	< 0.001	0.39 (0.26, 0.61)	< 0.001
5	0.52 (0.38, 0.72)	< 0.001	0.53 (0.39, 0.72)	< 0.001
6	0.55 (0.40, 0.76)	< 0.001	0.57 (0.42, 0.79)	< 0.001

	Prevalence of heavy-intensity infections (eggs per gram feces ≥ 400)			
Arm	Crude AMR (CI)	*P*-value	Adjusted AMR (CI)	*P*-value
1	0.38 (0.11, 1.27)	0.12	0.36 (0.11, 1.18)	0.09
2	0.13 (0.03, 0.61)	0.010	0.15 (0.03, 0.67)	0.01
3	0.32 (0.16, 0.64)	0.001	0.31 (0.16, 0.61)	< 0.001
4	0.24 (0.09, 0.60)	0.002	0.24 (0.09, 0.62)	0.003
5	0.32 (0.10, 1.00)	0.05	0.31 (0.10, 0.94)	0.04
6	0.35 (0.10, 1.20)	0.09	0.35 (0.11, 1.15)	0.08

	Intensity			
Arm	Crude AMR (CI)	*P*-value	Adjusted AMR (CI)	*P*-value
1	0.46 (0.19, 1.08)	0.07	0.46 (0.20, 1.08)	0.08
2	0.24 (0.08, 0.72)	0.01	0.24 (0.08, 0.73)	0.01
3	0.42 (0.24, 0.75)	0.003	0.42 (0.24, 0.74)	0.003
4	0.31 (0.16, 0.62)	< 0.001	0.31 (0.16, 0.62)	< 0.001
5	0.47 (0.20, 1.12)	0.09	0.46 (0.19, 1.11)	0.09
6	0.46 (0.19, 1.13)	0.09	0.47 (0.19, 1.15)	0.10

AMR = arithmetic mean ratio; PR = prevalence ratio. Adjusted PRs and AMRs control for age and gender.

**Table 3:** Praziquatel treatment coverage by arm and year

	Scheduled mass drug administration	SAC treated	SAC total	% SAC treated	Total population treated	Total population eligible	% Total population treated
Year 1							
Arm 1	CWT	2,788	3,980	70.1%	8,610	9,791	87.9%
Arm 2	CWT	2,572	3,444	74.7%	7,104	8,373	84.8%
Arm 3	CWT	2,505	3,615	69.3%	7,338	9,046	81.1%
Arm 4	SBT	7,696	9,103	84.5%	–	–	–
Arm 5	SBT	7,278	8,428	86.4%	–	–	–
Arm 6	SBT	7,395	8,911	83%	–	–	–
Year 2							
Arm 1	CWT	3,322	4,692	70.8%	9,228	10,858	85%
Arm 2	CWT	3,186	3,355	95%	7,750	8,736	88.7%
Arm 3	CWT	3,037	3,854	78.8%	7,951	8,930	89%
Arm 4	SBT	8,354	9,321	89.6%	–	–	–
Arm 5	SBT	7,630	8,475	90%	–	–	–
Arm 6	None	–	–	–	–	–	–
Year 3							
Arm 1	CWT*	8,540	10,255	83.3%	16,570	18,636	88.9%
Arm 2	SBT	8,236	8,907	92.5%	–	–	–
Arm 3	None	–	–	–	–	–	–
Arm 4	SBT	8,599	9,278	92.7%	–	–	–
Arm 5	None	–	–	–	–	–	–
Arm 6	SBT	8,580	9,285	92.4%	–	–	–
Year 4							
Arm 1	CWT*	10,254	12,051	85.1%	17,509	19,599	89.3%
Arm 2	SBT	7,527	8,413	89.5%	–	–	–
Arm 3	None	–	–	–	–	–	–
Arm 4	SBT	8,139	9,113	89.3%			
Arm 5	None	–	–	–	–	–	–
Arm 6	None	–	–	–	–	–	–

CWT = community-wide treatment; SAC = school-age children; SBT = school-based treatment.

* In years 3 and 4, SBT was provided along with CWT in arm 1.

**Figure 4.**
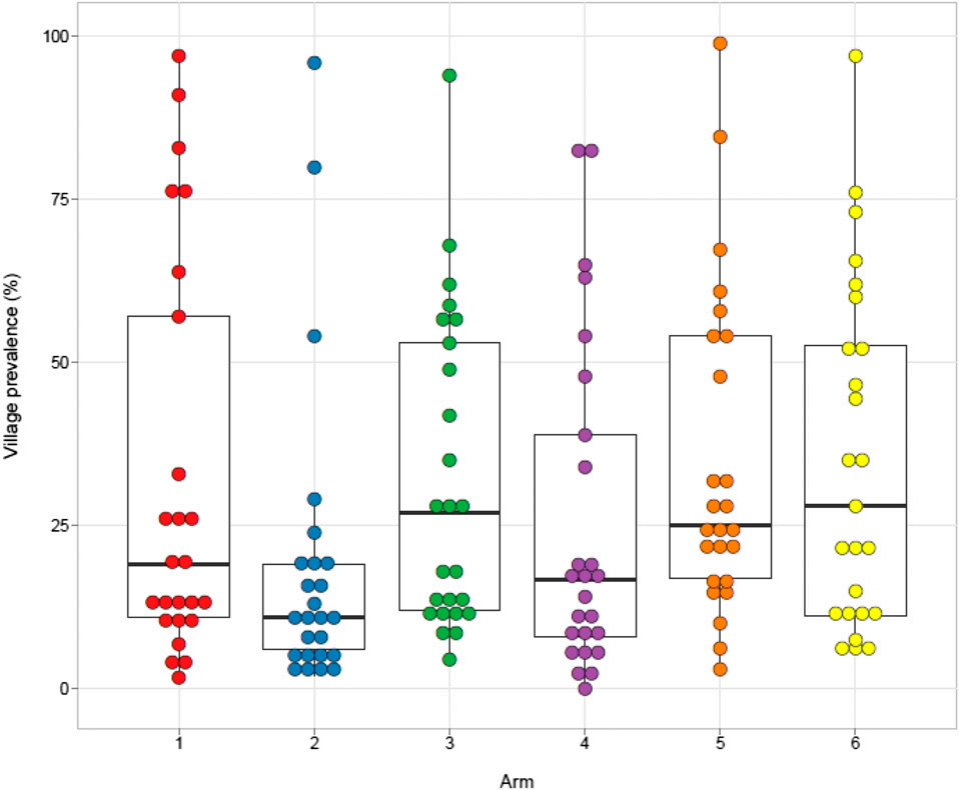
*Schistosoma mansoni* infection prevalence in 9- to 12-year-old children at year 5 final evaluation. Each dot represents an individual village. Bars represent arm median, boxes represent the 25th and 75th percentiles, and whiskers represent 5th and 95th percentiles. This figure appears in color at www.ajtmh.org.

**Figure 5.**
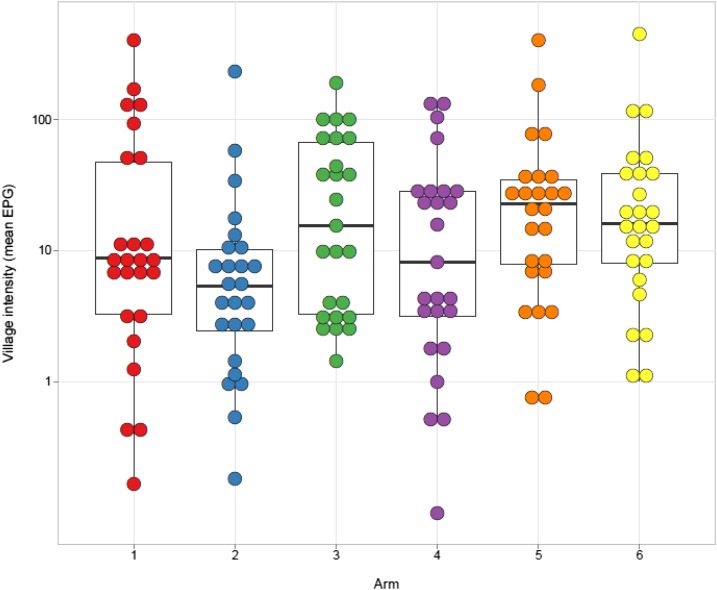
*Schistosoma mansoni* infection intensity in 9- to 12-year-old children at year 5 final evaluation. Each dot represents the mean eggs per gram feces in an individual village. Bars represent arm median, boxes represent the 25th and 75th percentiles, and whiskers represent 5th and 95th percentiles. This figure appears in color at www.ajtmh.org.

There was a larger than expected within-arm variability among villages in year 5 prevalence (Figure 4) and mean EPG (Figure 5). The interclass correlation coefficient for year 5 prevalence was 40% (95% CI 34–46%) and 19% (16–23%) for infection intensity. Nevertheless, the combined year 5 prevalence in villages in arms that received four rounds of treatment (arms 1, 2, and 4) was significantly lower than the combined year 5 prevalence in villages in arms that received only two rounds of treatment (arms 3, 5, and 6) over this period (PR = 0.68, 95% CI = 0.52–0.90, *P* = 0.008).

### First-year students and adults.

Only first-year students living in villages in arm 1 would have received MDA in year 4. Nevertheless, at the final evaluation in year 5, the prevalence for all arms and intensity in five of six arms were lower than those at baseline (Table 4), although this was only significantly different for prevalence in arms 1, 3, and 5 (arm 1: PR = 0.45, 95% CI = 0.26–0.79; arm 3: PR = 0.52, 95% CI = 0.28–0.97; and arm 5: PR = 0.56, 95% CI = 0.33–0.96, Table 5). As with 9–12-year-old students, the prevalence of heavy-intensity infections was reduced to < 5% in all arms, although none of the decreases were statistically significant.

**Table 4:** Prevalence and intensity summary statistics for first-year students at baseline and final evaluation by arm

		Year 1		Year 5	
Variable	Arm	*n*/*N* or *N*	% (CI), mean (CI), or median (range)	*n*/*N* or *N*	% (CI), mean (CI), or median (range)
Village prevalence (mean % infected, CI)	Arm 1	503/1,047	48.04 (31.76–64.32)	120/800	15.00 (7.51–22.49)
	Arm 2	134/657	20.40 (11.89–28.90)	103/726	14.19 (6.68–21.70)
	Arm 3	167/629	26.55 (17.70–35.40)	119/743	16.02 (7.76–24.27)
	Arm 4	256/742	34.50 (23.18–45.82)	153/687	22.27 (12.88–31.66)
	Arm 5	224/668	33.53 (23.11–43.95)	141/806	17.49 (10.13–24.86)
	Arm 6	401/982	40.84 (24.46–57.21)	192/979	19.61 (9.30–29.92)
Prevalence of heavy intensity infections (village mean % eggs per gram feces ≥ 400, CI)	Arm 1	69/1,047	6.59 (2.42–10.76)	28/800	3.50 (0.58–6.42)
	Arm 2	18/657	2.74 (0.00–5.50)	13/726	1.79 (0.00–3.76)
	Arm 3	23/629	3.66 (1.48–5.83)	19/743	2.56 (0.55–4.56)
	Arm 4	40/742	5.39 (1.15–9.63)	17/687	2.47 (0.74–4.21)
	Arm 5	30/668	4.49 (0.61–8.37)	16/806	1.99 (0.99–2.98)
	Arm 6	73/982	7.43 (1.54–13.33)	25/979	2.55 (0.00–5.52)
Village intensity (mean all participants, CI)	Arm 1	1,047	90.29 (43.38–137.20)	800	37.91 (8.52–67.29)
	Arm 2	657	35.27 (5.27–65.27)	726	32.63 (0.00–67.60)
	Arm 3	629	47.01 (22.80–71.22)	743	52.81 (0.00–112.69)
	Arm 4	742	67.71 (22.05–113.38)	687	35.09 (16.50–53.68)
	Arm 5	668	64.83 (25.32–104.34)	806	31.00 (16.06–45.94)
	Arm 6	982	83.76 (25.62–141.89)	979	50.87 (0.00–105.94)

Each arm consists of 25 villages.

**Table 5:** Comparisons of change in prevalence and intensity of *Schistosoma mansoni* infection between year 1 and year 5 for first-year students in each arm

	Prevalence			
Arm	Crude PR (CI)	*P-value*	Adjusted PR (CI)	*P-value*
1	0.46 (0.26, 0.83)	0.009	0.45 (0.26, 0.79)	0.005
2	0.67 (0.32, 1.36)	0.27	0.65 (0.34, 1.25)	0.20
3	0.57 (0.32, 1.03)	0.06	0.52 (0.28, 0.97)	0.04
4	0.68 (0.40, 1.17)	0.16	0.70 (0.40, 1.23)	0.22
5	0.57 (0.33, 0.99)	0.05	0.56 (0.33, 0.96)	0.03
6	0.60 (0.37, 0.98)	0.04	0.65 (0.40, 1.06)	0.09

	Prevalence of heavy intensity infections (EPG ≥ 400)			
Arm	Crude PR (CI)	*P-value*	Adjusted PR (CI)	*P-value*
1	0.53 (0.19, 1.51)	0.23	0.87 (0.30, 2.52)	0.80
2	0.65 (0.15, 2.88)	0.57	0.81 (0.19, 3.49)	0.78
3	0.70 (0.26, 1.86)	0.47	0.50 (0.19, 1.35)	0.17
4	0.46 (0.16, 1.31)	0.14	0.55 (0.18, 1.65)	0.28
5	0.44 (0.16, 1.19)	0.11	0.47 (0.17, 1.34)	0.16
6	0.34 (0.08, 1.39)	0.13	0.53 (0.16, 1.79)	0.30

	Intensity			
Arm	Crude AMR (CI)	*P-value*	Adjusted AMR (CI)	*P-value*
1	0.74 (0.29, 1.92)	0.54	0.76 (0.29, 2.00)	0.58
2	0.92 (0.24, 3.56)	0.91	0.86 (0.22, 3.38)	0.83
3	0.91 (0.29, 2.88)	0.87	0.97 (0.29, 3.18)	0.95
4	0.56 (0.24, 1.33)	0.19	0.52 (0.21, 1.26)	0.15
5	0.56 (0.25, 1.27)	0.17	0.53 (0.23, 1.23)	0.14
6	0.68 (0.27, 1.71)	0.41	0.70 (0.27, 1.82)	0.47

AMR = arithmetic mean ratio; PR = prevalence ratio; CI = 95% confidence interval. Adjusted PRs and AMRs control for age and gender.

Adult infection levels also changed from baseline to year 5, even in arms where CWT was not provided in year 4 (Table 6); prevalence was significantly lower in all arms (*P* < 0.0001), and the prevalence of heavy-intensity infections and overall infection intensity were significantly lower in five of six arms at year 5 than those at year 1 (Table 7). This was true even for villages that only ever received SBT where adults were never targeted for MDA. There was some benefit for having received CWT at some point as the year 5 infection prevalence in adults was lower in villages that had received at least two rounds of CWT (arms 1–3) than those that had only ever received SBT (arms 4–6; PR = 0.69, 95% CI = 0.50–0.95). The combined year 5 prevalence for villages in arms that received four rounds of treatment was not different from the combined year 5 prevalence for villages in arms that only had two total MDAs for first-year students (PR = 1.02, 95% CI – 0.71–1.47, *P* = 0.92) and adults (PR = 0.78, 95% CI = 0.57–1.07, *P* = 0.12, Table 4).

**Table 6:** Prevalence and intensity summary statistics for adults by arm at baseline and final evaluation

		Year 1		Year 5	
Variable	Arm	*n*/*N* or *N*	% (CI), mean (CI), or median (range)	*n*/*N* or *N*	% (CI), mean (CI), or median (range)
Village prevalence (mean % infected, CI)	Arm 1	559/1,193	46.86 (39.29–54.42)	148/1,244	11.90 (5.33–18.46)
	Arm 2	481/1,154	41.68 (33.27–50.10)	134/1,208	11.09 (6.29–15.90)
	Arm 3	475/1,164	40.81 (34.62–47.00)	138/1,121	12.31 (8.10–16.52)
	Arm 4	498/1,206	41.29 (33.56–49.03)	188/1,171	16.05 (9.76–22.35)
	Arm 5	595/1,164	51.12 (44.59–57.64)	217/1,202	18.05 (11.35–24.76)
	Arm 6	557/1,161	47.98 (40.40–55.55)	212/1,235	17.17 (13.52–20.81)
Prevalence of heavy-intensity infections (village mean % eggs per gram feces ≥ 400, CI)	Arm 1	38/1,193	3.19 (1.59–4.78)	11/1,244	0.88 (0.00–2.14)
	Arm 2	52/1,154	4.51 (2.12–6.90)	10/1,208	0.83 (0.00–1.72)
	Arm 3	42/1,164	3.61 (1.86–5.36)	6/1,121	0.54 (0.09–0.98)
	Arm 4	52/1,206	4.31 (1.75–6.88)	15/1,171	1.28 (0.10–2.46)
	Arm 5	68/1,164	5.84 (3.21–8.47)	21/1,202	1.75 (0.00–3.85)
	Arm 6	75/1,161	6.46 (3.15–9.77)	18/1,235	1.46 (0.77–2.14)
Village intensity (mean all participants, CI)	Arm 1	1,193	57.68 (40.93–74.43)	1,244	13.02 (1.79–24.25)
	Arm 2	1,154	68.24 (40.21–96.26)	1,208	15.62 (4.84–26.39)
	Arm 3	1,164	61.59 (42.50–80.67)	1,121	15.30 (6.36–24.23)
	Arm 4	1,206	60.12 (34.48–85.76)	1,171	22.51 (7.14–37.88)
	Arm 5	1,164	82.92 (59.30–106.53)	1,202	34.42 (0.00–72.16)
	Arm 6	1,161	81.04 (52.83–109.24)	1,235	25.05 (14.88–35.22)

Each arm consists of 25 villages.

**Table 7:** Comparisons of change in adult prevalence and intensity of *Schistosoma mansoni* infection between year 1 and year 5 for each arm

	Prevalence			
Arm	Crude PR (CI)	*P*-value	Adjusted PR (CI)	*P*-value
1	0.27 (0.15, 0.47)	< 0.001	0.25 (0.15, 0.45)	< 0.001
2	0.27 (0.17, 0.43)	< 0.001	0.26 (0.17, 0.42)	< 0.001
3	0.30 (0.21, 0.44)	< 0.001	0.30 (0.21, 0.44)	< 0.001
4	0.39 (0.25, 0.59)	< 0.001	0.37 (0.25, 0.56)	< 0.001
5	0.36 (0.24, 0.52)	< 0.001	0.38 (0.26, 0.54)	< 0.001
6	0.37 (0.28, 0.47)	< 0.001	0.38 (0.29, 0.49)	< 0.001

	Prevalence of heavy-intensity infections (eggs per gram feces ≥ 400)			
Arm	Crude AMR (CI)	*P*-value	Adjusted AMR (CI)	*P*-value
1	0.30 (0.07, 1.30)	0.11	0.27 (0.07, 1.05)	0.06
2	0.18 (0.05, 0.59)	0.005	0.18 (0.06, 0.61)	0.006
3	0.14 (0.06, 0.37)	< 0.001	0.17 (0.06, 0.46)	< 0.001
4	0.29 (0.10, 0.86)	0.03	0.25 (0.09, 0.71)	0.009
5	0.29 (0.08, 1.04)	0.06	0.29 (0.09, 0.99)	0.05
6	0.23 (0.11, 0.45)	< 0.001	0.19 (0.09, 0.45)	< 0.001

	Intensity			
Arm	Crude AMR (CI)	*P*-value	Adjusted AMR (CI)	*P*-value
1	0.24 (0.10, 0.58)	0.002	0.25 (0.10, 0.60)	0.002
2	0.23 (0.10, 0.50)	< 0.001	0.24 (0.11, 0.50)	< 0.001
3	0.24 (0.12, 0.46)	< 0.001	0.23 (0.12, 0.43)	< 0.001
4	0.37 (0.17, 0.81)	0.01	0.41 (0.19, 0.87)	0.02
5	0.41 (0.14, 1.25)	0.12	0.41 (0.14, 1.20)	0.10
6	0.32 (0.19, 0.54)	< 0.001	0.32 (0.19, 0.54)	< 0.001

AMR = arithmetic mean ratio; PR = prevalence ratio. Adjusted PRs and AMRs control for age and gender.

### Effect of removing “hotspot” villages.

**Figure 6.**
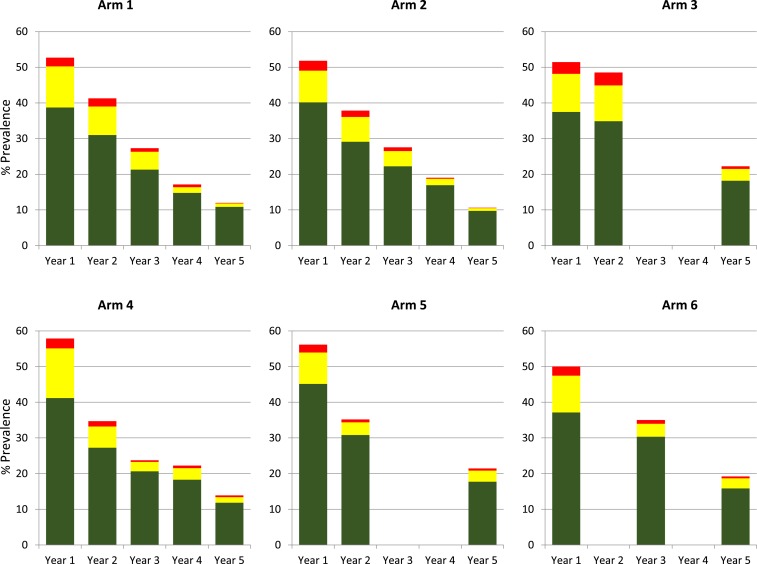
Overall mean prevalence and mean prevalence of light (green), moderate (yellow), and heavy (red) intensity infections by arm and year for 9- to 12-year-old children in villages not included in the “hot spot” area. Data for arm 1 represent 16 villages, arm 2 represent 19 villages, arm 3 represent 17 villages, arm 4 represent 17 villages, arm 5 represent 19 villages, and arm 6 represent 18 villages. This figure appears in color at www.ajtmh.org.

The high within-arm variability in prevalence (Figure 4) and infection intensity (Figure 5) at year 5 was not uniformly distributed across arms, and for some arms, villages fell into two distinct groups. When we mapped the villages that had persistent high prevalence or intensity, we noticed that they were geographically clustered and have reported that they may represent geographic “hotspots” that respond to MDA differently than most of the villages in the study.^10^ Re-evaluation of the effect of the MDA strategies after removing the 6–9 hotspot villages per arm from the analysis (Figure 6) resulted in a significant reduction in prevalence (*P* < 0.001) and intensity (*P* ≤ 0.002) of *S. mansoni* infections in 9–12-year-olds over the time of the study within each arm. There were also significant differences in 5th year prevalence (Supplemental Table 3) and overall prevalence reduction from year 1 to year 5 (Supplemental Table 4) between arms 2 (*P* ≤ 0.003 and *P* ≤ 0.017, respectively) and 4 (*P* ≤ 0.007 and *P* ≤ 0.02, respectively) compared to arms 3, 5, or 6. Furthermore, although there was only roughly a 50% decrease in prevalence from year 1 to year 5 for most arms when all villages were included (Figure 3), when hotspot villages were removed, there was up to an 80% decrease in prevalence in the villages that received four rounds of treatment (Figure 6 arms 1, 2, and 4). This decrease was significantly greater (PR = 0.57, 95% CI = 0.42–0.79, *P* < 0.001) than the change seen in the villages that received only two rounds of treatment (arms 3, 5, and 6). The prevalence and intensity of infection at year 5 was also significantly lower for the combined arms receiving four rounds of MDA than those that had only received two (PR = 0.54, 95% CI = 0.41–0.72, *P* < 0.001; AMR = 0.43, 95% CI = 0.27–0.67, *P* < 0.001). For all arms, the prevalence of heavy-intensity infections after hotspot village removal was less than 1%, the current WHO target for elimination of schistosomiasis as a public health problem.

## DISCUSSION

The distribution of MDA for schistosomiasis has increased substantially since the adoption of World Health Assembly Resolution 54.19 in 2001, with coverage levels for SAC approaching the target goal of 75%.^11^ However, despite generous donations from pharmaceutical companies, government agencies, and private foundations, the quantity of praziquantel available still falls well short of the global demand, especially for adult populations. The need to use limited resources as efficiently and effectively as possible was part of the stimulus for the SCORE projects comparing different frequencies or recipient populations of praziquantel MDA. We found that both annual or biennial MDA distributed in schools (SBT) or at the community level (CWT) had roughly similar benefits, with significant decreases in overall prevalence and prevalence of heavy-intensity (≥ 400 EPG) infections in school-aged (9–12-year-old) children after two to four rounds of MDA. Although there were no statistically significant differences between individual arms, by analyzing combined data from children aged 9 to 12 years, we found that the arms that received a total of four rounds of MDA had significantly lower prevalence at the final evaluation than the compiled data from arms that only received two rounds of MDA during the study.

The finding of statistically significant decreases in prevalence over time within arms but no statistical differences between individual arms is similar to the results for SCORE evaluations we performed in Kenyan villages with 10–24% initial prevalence^12^ and parallel studies in villages with > 25% initial *S. mansoni* and *S. haematobium* prevalence conducted in Tanzania and Mozambique, respectively.^13,14^ The lack of statistical differences in prevalence in 9–12-year-olds between individual arms may in part reflect a much higher design effect (37.87, 95% CI 32.41, 43.32) than anticipated when the study was planned. Although schistosomiasis is known to be a highly focal disease, the very large interclass correlation (40%) observed in this study practically precludes the ability to detect significant differences between individual arms. Thus, should similar studies be performed in the future; it will likely be necessary to have a much higher number of villages per arm, fewer arms, evaluations at the village-level, or a completely different approach.

One surprising result from the study was the dramatic reduction in infection prevalence and intensity in the adult population, even in arms in which only SBT was administered. There were also reductions in the infection levels of first-year students at year 5 compared to year 1, although most of these changes were not statistically significant. As with the adults, the reductions were similar for SBT only arms, where these individuals would not have received treatment before their entry in to school, compared with CWT arms. The reduced infections in the non-SAC study participants at year 5, even in SBT arms, may reflect decreases in the force of transmission as a result of lower levels of SAC infection, leading to fewer eggs excreted into the environment and a smaller number of snails becoming infected. This could contribute to fewer infections because of decreased concentrations of cercariae in the waters where individuals are exposed. The SCORE study in Mozambique observed a similar reduction in adult infections with *S. haematobium*, and the investigators also attributed it to reduced rates of transmission.^14^

These data are also consistent with host immune responses contributing to earlier worm death. Adult *S. mansoni* worms are estimated to have a 5.7 to 10.5-year life span.^15^ Thus, the 60–75% reduction of infection we observed in adults not receiving treatment is greater than would be expected even if transmission rates were drastically reduced. In reinfection studies, older adolescents and adults have changes in their immune responses that confer greater resistance to reinfection than children and early adolescents.^16–18^ Although these immune responses have previously been defined in relation to reducing infection with new cercariae, our data suggest they could also affect existing worms. Additional investigations will be needed to confirm or refute this hypothesis. Nevertheless, MDA benefits for non-treated groups in both *S. mansoni*– and *S. haematobium*–endemic areas is a welcome finding for schistosomiasis control programs.

In addition to the higher than expected design effect, other limitations of our study included the lower than targeted coverage rates, especially among SAC in CWT arms during the first two years. Once we identified these shortfalls, we increased our training of community drug distributors as well as our communication efforts,^19,20^ resulting in better coverage rates in the last 2 years. We were also not always able to recruit the desired sample size to measure infection, especially in the early years of the study and among first-year students as there were fewer potential participants of this age. However, we do not anticipate that this had a large influence on study outcomes given the high degree of correlation within villages that we observed.

This study confirmed the impact of MDA as shown by reduction in prevalence and intensity across all arms and in all age-groups. Although greater within-arm variability than expected limited the ability to see significant differences between arms; the very similar patterns of infection level over time in arms that received four rounds of CWT (arm 1), SBT (arm 4), or a combination of the two (arm 2) suggest that SBT, which typically costs less and is easier to distribute, has comparable benefits as CWT. The surprising finding that SBT also has benefits for non-targeted age-groups reinforces the conclusion that SBT may be adequate for most ongoing control programs. Nevertheless, because all individuals who are infected likely suffer some health detriment,^21^ inclusion of adults for 1 or two rounds of treatment followed by SBT (such as employed in arm 2) may be the strategy with the best cost to benefit ratio. However, because preschool-aged children can also have high levels of infection and may be particularly susceptible to harm caused by schistosomiasis, control programs should also include efforts to treat this age-group. Furthermore, in settings where it is appropriate to move from control to elimination of transmission, it will become important to treat all ages to remove any refugia of infection. Finally, for control programs to use MDA as effectively as possible, it is also important to understand the impact of local disease epidemiology, such as the presence of infection hotspots.^10^

## Supplemental tables

Supplemental materials
