# Supplementary material for: Comparison of School-Based and Community-Wide Mass Drug Administration for Schistosomiasis Control in an Area of Western Kenya with High Initial Schistosoma mansoni Infection Prevalence: A Cluster Randomized Trial
Source: Am J Trop Med Hyg. 2019 Dec 2;102(2):318–27. doi: 10.4269/ajtmh.19-0626 (PMC7008345; doi:10.4269/ajtmh.19-0626)
Supplement: Supplementary file 1 [file tpmd190626.SD1.doc]

Supplemental Table 1. Numbers, prevalence, and infection intensity of 9-12 year old children in years 2, 3, and 4.

|  |  | Year 2 |  | Year 3 |  | Year 4 |  |
| --- | --- | --- | --- | --- | --- | --- | --- |
| Variable | Arm | n/N or N | % (CI), mean (CI), or median (range) | n/N or N | % (CI), mean (CI), or median (range) | n/N or N | % (CI), mean (CI), or median (range) |
| Prevalence | Arm 1 | 961/1658 | 57.96 (47.84 - 68.08) | 990/2085 | 47.48 (36.19 - 58.78) | 882/2327 | 37.90 (27.13 - 48.68) |
|  | Arm 2 | 823/1873 | 43.94 (34.42 - 53.46) | 841/2143 | 39.24 (28.95 - 49.54) | 723/2457 | 29.43 (20.01 - 38.84) |
|  | Arm 3 | 988/1765 | 55.98 (47.56 - 64.39) |  |  |  |  |
|  | Arm 4 | 800/1784 | 44.84 (34.60 - 55.08) | 679/1996 | 34.02 (24.02 - 44.02) | 797/2429 | 32.81 (22.08 - 43.54) |
|  | Arm 5 | 885/1876 | 47.17 (36.47 - 57.88) |  |  |  |  |
|  | Arm 6 |  |  | 1101/2174 | 50.64 (40.37 - 60.92) |  |  |
|  |  |  |  |  |  |  |  |
| Prevalence | Arm 1 | 143/1658 | 8.62 (3.91 - 13.34) | 123/2085 | 5.90 (2.18 - 9.62) | 72/2327 | 3.09 (1.37 - 4.82) |
| (EPG ≥ 400) | Arm 2 | 114/1873 | 6.09 (0.19 - 11.98) | 106/2143 | 4.95 (0.55 - 9.35) | 40/2457 | 1.63 (0.24 - 3.02) |
|  | Arm 3 | 111/1765 | 6.29 (3.21 - 9.37) |  |  |  |  |
|  | Arm 4 | 49/1784 | 2.75 (1.31 - 4.18) | 41/1996 | 2.05 (0.50 - 3.61) | 44/2429 | 1.81 (0.70 - 2.93) |
|  | Arm 5 | 57/1876 | 3.04 (0.80 - 5.28) |  |  |  |  |
|  | Arm 6 |  |  | 71/2174 | 3.27 (1.08 - 5.45) |  |  |
|  |  |  |  |  |  |  |  |
| Intensity | Arm 1 | 823 | 154.26 (73.23 - 235.29) | 679 | 97.49 (63.80 - 131.18) | 2327 | 0 (0 - 3104) |
| (mean, all | Arm 2 | 988 | 141.62 (104.56 - 178.68) | 1101 | 105.80 (73.79 - 137.82) | 2457 | 0 (0 - 1976) |
| participants) | Arm 3 | 800 | 97.02 (77.53 - 116.51) |  |  |  |  |
|  | Arm 4 | 885 | 96.46 (59.28 - 133.64) | 2085 | 0 (0 - 1008) | 2429 | 0 (0 - 2160) |
|  | Arm 5 | 1658 | 8 (0 - 1008) |  |  |  |  |
|  | Arm 6 |  |  | 2143 | 0 (0 - 1008) |  |  |

CI = 95% confidence interval; EPG = eggs per gram feces.

Each arm consists of 25 villages.

Supplemental Table 2. Comparing changes from year 1 to year 5 between arms, prevalence and intensity, 9-12 year olds.

|  | Prevalence |  |  |  | Intensity |  |  |  |
| --- | --- | --- | --- | --- | --- | --- | --- | --- |
| Comparison | Crude PR (CI) | p | Adjusted PR (CI) | p | Crude AMR (CI) | p | Adjusted AMR (CI) | p |
| arm 2 vs. arm 1 | 0.66 (0.35, 1.25) | 0.21 | 0.68 (0.37, 1.24) | 0.21 | 0.51 (0.13, 2.10) | 0.35 | 0.51 (0.12, 2.11) | 0.36 |
| arm 3 vs. arm 1 | 1.04 (0.62, 1.72) | 0.89 | 1.20 (0.73, 1.96) | 0.47 | 0.92 (0.33, 2.57) | 0.88 | 0.92 (0.33, 2.56) | 0.87 |
| arm 4 vs. arm 1 | 0.83 (0.47, 1.47) | 0.52 | 0.80 (0.45, 1.42) | 0.44 | 0.68 (0.23, 2.03) | 0.49 | 0.68 (0.23, 2.03) | 0.49 |
| arm 5 vs. arm 1 | 1.03 (0.62, 1.70) | 0.92 | 1.07 (0.66, 1.76) | 0.78 | 1.02 (0.30, 3.47) | 0.97 | 1.01 (0.30, 3.43) | 0.99 |
| arm 6 vs. arm 1 | 1.08 (0.65, 1.80) | 0.76 | 1.16 (0.70, 1.91) | 0.56 | 1.01 (0.29, 3.48) | 0.98 | 1.02 (0.30, 3.53) | 0.97 |
| arm 3 vs. arm 2 | 1.56 (0.86, 2.81) | 0.14 | 1.76 (1.01, 3.06) | 0.045 | 1.80 (0.51, 6.32) | 0.36 | 1.79 (0.51, 6.30) | 0.37 |
| arm 4 vs. arm 2 | 1.25 (0.65, 2.38) | 0.50 | 1.17 (0.62, 2.20) | 0.62 | 1.32 (0.36, 4.92) | 0.68 | 1.32 (0.35, 4.93) | 0.68 |
| arm 5 vs. arm 2 | 1.54 (0.86, 2.78) | 0.15 | 1.58 (0.90, 2.74) | 0.11 | 1.99 (0.48, 8.24) | 0.34 | 1.97 (0.47, 8.17) | 0.35 |
| arm 6 vs. arm 2 | 1.63 (0.91, 2.93) | 0.10 | 1.70 (0.97, 2.98) | 0.06 | 1.97 (0.47, 8.25) | 0.35 | 1.99 (0.47, 8.40) | 0.35 |
| arm 4 vs. arm 3 | 0.80 (0.47, 1.35) | 0.41 | 0.67 (0.39, 1.12) | 0.13 | 0.74 (0.30, 1.79) | 0.50 | 0.74 (0.30, 1.80) | 0.51 |
| arm 5 vs. arm 3 | 0.99 (0.63, 1.55) | 0.97 | 0.90 (0.58, 1.38) | 0.62 | 1.11 (0.39, 3.14) | 0.85 | 1.10 (0.39, 3.13) | 0.86 |
| arm 6 vs. arm 3 | 1.04 (0.67, 1.64) | 0.85 | 0.97 (0.63, 1.50) | 0.88 | 1.10 (0.38, 3.15) | 0.86 | 1.11 (0.39, 3.21) | 0.84 |
| arm 5 vs. arm 4 | 1.24 (0.74, 2.08) | 0.42 | 1.35 (0.79, 2.28) | 0.27 | 1.50 (0.50, 4.56) | 0.47 | 1.49 (0.49, 4.51) | 0.48 |
| arm 6 vs. arm 4 | 1.31 (0.78, 2.20) | 0.32 | 1.45 (0.85, 2.47) | 0.17 | 1.49 (0.49, 4.58) | 0.48 | 1.51 (0.49, 4.63) | 0.48 |
| arm 6 vs. arm 5 | 1.06 (0.68, 1.65) | 0.81 | 1.08 (0.70, 1.68) | 0.73 | 0.99 (0.29, 3.45) | 0.99 | 1.01 (0.29, 3.54) | 0.98 |

PR = prevalence ratio; CI = 95% confidence interval; AMR = arithmetic mean ratio.

Adjusted PRs and AMRs control for age and sex.

Supplemental Table 3. Comparing changes at year 5 between arms, prevalence and intensity, 9-12 year olds, hotspot villages removed.

|  | Prevalence |  |  |  | Intensity |  |  |  |
| --- | --- | --- | --- | --- | --- | --- | --- | --- |
| Comparison | Crude PR (CI) | p | Adjusted PR (CI) | p | Crude AMR (CI) | p | Adjusted AMR (CI) | p |
| arm 2 vs. arm 1 | 0.69 (0.42, 1.14) | 0.15 | 0.69 (0.42, 1.14) | 0.15 | 0.61 (0.28, 1.33) | 0.22 | 0.61 (0.28, 1.31) | 0.20 |
| arm 3 vs. arm 1 | 1.36 (0.78, 2.38) | 0.28 | 1.42 (0.82, 2.46) | 0.21 | 1.97 (0.79, 4.91) | 0.15 | 1.97 (0.79, 4.94) | 0.15 |
| arm 4 vs. arm 1 | 0.73 (0.43, 1.24) | 0.25 | 0.69 (0.41, 1.19) | 0.18 | 1.08 (0.44, 2.64) | 0.86 | 1.07 (0.44, 2.62) | 0.88 |
| arm 5 vs. arm 1 | 1.50 (0.92, 2.46) | 0.11 | 1.50 (0.91, 2.46) | 0.11 | 2.32 (1.01, 5.33) | 0.05 | 2.30 (1.01, 5.28) | 0.05 |
| arm 6 vs. arm 1 | 1.41 (0.85, 2.36) | 0.19 | 1.43 (0.86, 2.36) | 0.17 | 1.82 (0.82, 4.06) | 0.14 | 1.79 (0.80, 3.99) | 0.16 |
| arm 3 vs. arm 2 | 1.97 (1.22, 3.18) | 0.006 | 2.05 (1.27, 3.30) | 0.003 | 3.22 (1.59, 6.49) | 0.001 | 3.26 (1.62, 6.54) | < 0.001 |
| arm 4 vs. arm 2 | 1.06 (0.68, 1.65) | 0.81 | 1.00 (0.63, 1.59) | 1.00 | 1.77 (0.90, 3.46) | 0.10 | 1.77 (0.90, 3.45) | 0.10 |
| arm 5 vs. arm 2 | 2.17 (1.45, 3.25) | < 0.001 | 2.16 (1.43, 3.27) | < 0.001 | 3.78 (2.09, 6.86) | < 0.001 | 3.80 (2.13, 6.79) | < 0.001 |
| arm 6 vs. arm 2 | 2.04 (1.34, 3.12) | < 0.001 | 2.05 (1.35, 3.13) | < 0.001 | 2.97 (1.72, 5.14) | < 0.001 | 2.95 (1.72, 5.07) | < 0.001 |
| arm 4 vs. arm 3 | 0.54 (0.32, 0.90) | 0.02 | 0.49 (0.29, 0.82) | 0.007 | 0.55 (0.24, 1.25) | 0.15 | 0.54 (0.24, 1.24) | 0.15 |
| arm 5 vs. arm 3 | 1.10 (0.68, 1.78) | 0.69 | 1.05 (0.65, 1.70) | 0.83 | 1.18 (0.55, 2.52) | 0.68 | 1.17 (0.55, 2.48) | 0.69 |
| arm 6 vs. arm 3 | 1.04 (0.63, 1.70) | 0.89 | 1.00 (0.62, 1.62) | 0.99 | 0.92 (0.45, 1.91) | 0.83 | 0.91 (0.44, 1.88) | 0.79 |
| arm 5 vs. arm 4 | 2.05 (1.32, 3.20) | 0.002 | 2.16 (1.36, 3.43) | 0.001 | 2.14 (1.03, 4.47) | 0.04 | 2.15 (1.04, 4.44) | 0.04 |
| arm 6 vs. arm 4 | 1.93 (1.22, 3.06) | 0.005 | 2.06 (1.28, 3.29) | 0.003 | 1.68 (0.84, 3.39) | 0.15 | 1.67 (0.83, 3.36) | 0.15 |
| arm 6 vs. arm 5 | 0.94 (0.62, 1.43) | 0.77 | 0.95 (0.62, 1.45) | 0.82 | 0.78 (0.42, 1.47) | 0.45 | 0.78 (0.42, 1.44) | 0.42 |

PR = prevalence ratio; CI = 95% confidence interval; AMR = arithmetic mean ratio.

Adjusted PRs and AMRs control for age and sex.

Supplemental Table 4. Comparing changes from year 1 to year 5 between arms, prevalence and intensity, 9-12 year olds, hotspot villages removed.

|  | Prevalence |  |  |  | Intensity |  |  |  |
| --- | --- | --- | --- | --- | --- | --- | --- | --- |
| Comparison | Crude PR (CI) | p | Adjusted PR (CI) | p | Crude AMR (CI) | p | Adjusted AMR (CI) | p |
| arm 2 vs. arm 1 | 0.70 (0.40, 1.22) | 0.21 | 0.73 (0.42, 1.27) | 0.26 | 0.65 (0.26, 1.65) | 0.36 | 0.64 (0.25, 1.64) | 0.36 |
| arm 3 vs. arm 1 | 1.31 (0.70, 2.45) | 0.40 | 1.39 (0.75, 2.57) | 0.29 | 1.59 (0.57, 4.48) | 0.38 | 1.59 (0.56, 4.53) | 0.38 |
| arm 4 vs. arm 1 | 0.72 (0.40, 1.33) | 0.30 | 0.68 (0.37, 1.25) | 0.22 | 1.00 (0.35, 2.85) | 1.00 | 1.00 (0.35, 2.88) | 1.00 |
| arm 5 vs. arm 1 | 1.35 (0.78, 2.35) | 0.28 | 1.37 (0.79, 2.39) | 0.26 | 2.02 (0.72, 5.68) | 0.18 | 2.00 (0.70, 5.69) | 0.19 |
| arm 6 vs. arm 1 | 1.38 (0.78, 2.42) | 0.27 | 1.39 (0.79, 2.44) | 0.25 | 1.75 (0.69, 4.44) | 0.24 | 1.75 (0.68, 4.51) | 0.24 |
| arm 3 vs. arm 2 | 1.87 (1.09, 3.21) | 0.02 | 1.91 (1.12, 3.26) | 0.017 | 2.46 (1.03, 5.88) | 0.04 | 2.47 (1.03, 5.96) | 0.04 |
| arm 4 vs. arm 2 | 1.03 (0.62, 1.73) | 0.90 | 0.94 (0.56, 1.59) | 0.82 | 1.55 (0.64, 3.75) | 0.33 | 1.56 (0.64, 3.80) | 0.33 |
| arm 5 vs. arm 2 | 1.93 (1.23, 3.04) | 0.004 | 1.89 (1.19, 3.00) | 0.007 | 3.12 (1.30, 7.46) | 0.01 | 3.11 (1.29, 7.49) | 0.01 |
| arm 6 vs. arm 2 | 1.97 (1.24, 3.13) | 0.004 | 1.91 (1.19, 3.06) | 0.007 | 2.69 (1.27, 5.71) | 0.010 | 2.73 (1.28, 5.82) | 0.01 |
| arm 4 vs. arm 3 | 0.55 (0.31, 1.00) | 0.05 | 0.49 (0.27, 0.88) | 0.02 | 0.63 (0.23, 1.70) | 0.36 | 0.63 (0.23, 1.71) | 0.37 |
| arm 5 vs. arm 3 | 1.03 (0.60, 1.76) | 0.91 | 0.99 (0.58, 1.68) | 0.96 | 1.27 (0.48, 3.39) | 0.63 | 1.26 (0.47, 3.38) | 0.65 |
| arm 6 vs. arm 3 | 1.05 (0.61, 1.81) | 0.86 | 1.00 (0.58, 1.71) | 1.00 | 1.10 (0.46, 2.63) | 0.84 | 1.10 (0.45, 2.67) | 0.83 |
| arm 5 vs. arm 4 | 1.87 (1.12, 3.11) | 0.02 | 2.01 (1.19, 3.38) | 0.009 | 2.02 (0.75, 5.44) | 0.17 | 1.99 (0.74, 5.41) | 0.18 |
| arm 6 vs. arm 4 | 1.90 (1.13, 3.20) | 0.02 | 2.03 (1.20, 3.44) | 0.008 | 1.74 (0.72, 4.23) | 0.22 | 1.75 (0.71, 4.29) | 0.22 |
| arm 6 vs. arm 5 | 1.02 (0.64, 1.61) | 0.94 | 1.01 (0.63, 1.62) | 0.96 | 0.86 (0.36, 2.07) | 0.74 | 0.88 (0.36, 2.13) | 0.77 |

PR = prevalence ratio; CI = 95% confidence interval; AMR = arithmetic mean ratio.

Adjusted PRs and AMRs control for age and sex.
